# Institutional Pharmacists and the "B.P."

**Published:** 1915-03-06

**Authors:** 


### Institutional Pharmacists and the "B.P."

A meeting of the Public Pharmacists' and Dispensers' Association was held at St. Bride Institute, Ludgate Circus, E.C., on February 24. There was a good attendance of members, Mr. J. Hassall France being in the chair, supported by Messrs. Welford, Lindsey, Lindsay, Gibbons, Jenkin, Moore, Heworth, Gibson (hon. secretary and treasurer), and others. The evening was devoted to two "Short Papers" by members.

Mr. A. J. Gibbons, St. Thomas's Hospital, gave an interesting paper on "Red Cross Work," mentioning more especially the duties devolving upon the pharmacist of a Voluntary Aid Detachment of the Red Cross Society. In the course of his paper he mentioned that fifty men of his detachment had already volunteered and were on active service in France. Incidentally it was noted that the pharmacist, whilst he obtains no rank in the R.A.M.C. *qua* pharmacist, is recognised by the Red Cross Society, who class him as an officer, subordinate to the medical officer of his detachment.

Mr. A. H. Jenkin, M.P.S., opened a discussion on the British Pharmacopœia, 1914. The large increase in the strengths of the strophanthus and opium tinctures were noted; many preparations have also been weakened—viz., tinctures of nux vomica, belladonna, digitalis, colchicum, etc. Many preparations which are in frequent use by medical practitioners should have been included, notably "chlorodyne." Tr. chlorof. et morphin. co. has never been largely prescribed, medical men preferring a preparation more on the lines of the 1885 Pharmacopœia. Hyoscyamus preparations should, in Mr. Jenkin's opinion, have been standardised. Whilst the metric system would probably be largely used by pharmacists in their laboratories, medical men would no doubt continue to "think" in grains and ounces, and the fact that the general public had become used to the domestic terms "tablespoon," "teaspoon," etc., would delay any extended use of metric terms outside of the dispensary. The new stipulation regarding "unusually large doses"—in which the pharmacist "is expected to satisfy himself that the prescriber's intention had been correctly interpreted"—came in for some discussion, many members expressing the difficulties of ascertaining the prescriber's intention in certain cases.

Mr. Jenkin further thought that the modern method of "milling" ointments should have had a place in the British Pharmacopœia. With regard to injections—strychnine being .75 per cent. will cause some "mental arithmetic" to ascertain the liquid equivalent to fractions of a grain of the alkaloid. A very full discussion ensued, in which most of those present took part. It was stated that regarding "unusually large doses" the preferable plan would have been to stipulate that no doses in excess of those given in the British Pharmacopœia should be dispensed unless the medical practitioner's initials were placed against the quantity ordered on the prescription.

A "10-mil." conical measure glass was exhibited and attention drawn to the similarity between this and a minim measure. Although pharmacists would readily distinguish between the two, it was agreed that if these metric measures were inadvertently issued to the hospital wards, members of the nursing staff might possibly mistake the "10 mils." for 10 minims. In the case of opium, etc., this would end in disastrous results. Hospital Pharmacopœias will require some revision, but as a whole no very great changes need be made.

Messrs. Hamilton and Ritchie (of Belfast) were elected members of the Association.

